# Consumption of *Bt* Maize Pollen Expressing Cry1Ab or Cry3Bb1 Does Not Harm Adult Green Lacewings, *Chrysoperla carnea* (Neuroptera: Chrysopidae)

**DOI:** 10.1371/journal.pone.0002909

**Published:** 2008-08-06

**Authors:** Yunhe Li, Michael Meissle, Jörg Romeis

**Affiliations:** Agroscope Reckenholz-Tänikon Research Station ART, Zurich, Switzerland; University of Kentucky, United States of America

## Abstract

Adults of the common green lacewing, *Chrysoperla carnea* (Stephens) (Neuroptera: Chrysopidae), are prevalent pollen-consumers in maize fields. They are therefore exposed to insecticidal proteins expressed in the pollen of insect-resistant, genetically engineered maize varieties expressing Cry proteins derived from *Bacillus thuringiensis* (*Bt*). Laboratory experiments were conducted to evaluate the impact of Cry3Bb1 or Cry1Ab-expressing transgenic maize (MON 88017, Event Bt176) pollen on fitness parameters of adult *C. carnea*. Adults were fed pollen from *Bt* maize varieties or their corresponding near isolines together with sucrose solution for 28 days. Survival, pre-oviposition period, fecundity, fertility and dry weight were not different between *Bt* or non-*Bt* maize pollen treatments. In order to ensure that adults of *C. carnea* are not sensitive to the tested toxins independent from the plant background and to add certainty to the hazard assessment, adult *C. carnea* were fed with artificial diet containing purified Cry3Bb1 or Cry1Ab at about a 10 times higher concentration than in maize pollen. Artificial diet containing *Galanthus nivalis* agglutinin (GNA) was included as a positive control. No differences were found in any life-table parameter between Cry protein containing diet treatments and control diet. However, the pre-oviposition period, daily and total fecundity and dry weight of *C. carnea* were significantly negatively affected by GNA-feeding. In both feeding assays, the stability and bioactivity of Cry proteins in the food sources as well as the uptake by *C. carnea* was confirmed. These results show that adults of *C. carnea* are not affected by *Bt* maize pollen and are not sensitive to Cry1Ab and Cry3Bb1 at concentrations exceeding the levels in pollen. Consequently, *Bt* maize pollen consumption will pose a negligible risk to adult *C. carnea*.

## Introduction

Genetically engineered (GE) maize varieties expressing Cry proteins from the bacterium *Bacillus thuringiensis* (*Bt*) are the most widely grown insect-resistant transgenic plants to date. First commercialized in 1996, *Bt* maize was grown in 13 countries on a total of over 35 million hectares (>24% of maize area worldwide) in 2007 [1], [2]. Most *Bt* maize varieties are protected against lepidopteran pests, i.e. the European corn borer, *Ostrinia nubilalis* (Hübner) (Lepidoptera: Crambidae), and other stemborers, by expression of Lepidoptera-specific Cry proteins such as Cry1Ab. More recently, varieties have become commercially available in the USA that express Coleoptera-active toxins such as Cry3Bb1 for the control of corn rootworms (*Diabotica* spp.; Coleoptera: Chrysomelidae). These two groups of herbivores are major pests of maize worldwide and cause substantial economic losses to maize farmers every year. Most maize growers rely on traditional crop protection practices to manage them, including cultural, biological or chemical methods. While stem borers are often left uncontrolled by insecticides due to difficulties in the timing of their application, insecticides used against *Diabrotica* spp. in the USA alone comprise 25–30% of global total insecticide used in maize [2]. GE maize offers a new effective alternative with economic and environmental advantages over using conventional insecticides to manage these maize pests. In particular Coleoptera-active *Bt* maize shows a greater potential for insecticide reduction in the near future [2].

One of the risks associated with the growing of insect-resistant GE maize varieties is their potential to adversely affect non-target organisms. The assessment of such effects is thus part of the environmental risk assessment that is conducted prior to commercialization of any novel GE variety. Natural enemies of pest arthropods are of particular interest as they provide an important ecological function, i.e. the regulation of herbivores, and thus contribute to a sustainable agro-ecosystem [3]. When assessing the risk to non-target arthropods, both the exposure to the plant-expressed insecticidal protein and the hazard of being exposed need to be considered [4], [5].

Maize produces large amounts of pollen, i.e. each maize tassel produces up to 50 million pollen grains over a period of 5–8 days [6], [7]. During anthesis, maize pollen is an abundant and nutritious food resource [8], [9]. Consequently, it is used as food by a number of insects, including many predatory species [10]–[13]. In case of the Cry protein being expressed in the pollen of *Bt* maize varieties, pollen should be taken into account as an important route by which non-target organisms could be exposed to the insecticidal protein [3], [14], [15].

The common green lacewing, *Chrysoperla carnea* (Stephens) (Neuroptera: Chrysopidae), is considered as an important natural predator of insect pests due to the fact that it has a wide geographic distribution, occurs in many different crop and non-crop habitats, is tolerant to certain pesticides, and has a voracious larval feeding capacity [16]–[18]. The predatory larval stages feed preferentially on aphids but may consume a wide range of soft-bodied arthropods. However in *Bt* maize, exposure of the larvae to the *Bt* toxins is considered to be relatively low as aphids contain no or only trace amounts of the toxins due to the fact that they feed on the phloem sap which does not contain Cry proteins [19], [20]. *C. carnea* adults on the other hand are not predacious but feed primarily on pollen, nectar and honeydew [21]–[24]. A preliminary laboratory study revealed that maize pollen is readily utilized by adult *C. carnea* (Li et al. unpublished). *Bt* maize varieties are known to express the Cry proteins in pollen. Therefore, adult *C. carnea* will ingest the toxin by consuming maize pollen during anthesis. This was confirmed by Obrist et al. [15] who showed that no or very little Cry1Ab toxin was detected in adult *C. carnea* collected in *Bt* maize (Event Bt176) fields before and after pollen shed, but substantial toxin levels were present in insects caught during anthesis. *Bt* maize based on Event Bt176 contains considerable amounts of Cry1Ab in the pollen that are in the order of magnitude of leaf material [14], [25]. This event, however, has in the meantime been removed from the market. The currently grown Cry1Ab-expressing maize varieties (events MON 810 or Bt 11), in contrast, contain substantially lower levels of toxin in the pollen (i.e. more than 100 times lower than in green leaf tissue; Dutton et al. [14]). *Bt* toxin concentrations in pollen of today's corn-rootworm (*Diabrotica* spp.)-resistant *Bt* maize events MON 863 and MON 88017, however, are much closer to the concentrations in leaves [26]–[28].

Previous work on *Bt* maize effects on *C. carnea* has focused on the larval stages and a number of studies have now revealed that the insects do not appear to be directly affected by Cry1Ab [20], [29]–[31], contradicting an earlier report of direct toxicity [32]. Despite the fact that adult lacewings are likely to be directly exposed (through pollen feeding) to higher *Bt* concentrations than the larval stage, no studies have been conducted with adults so far. Likely, the different feeding biology of adults compared to larvae may also be reflected in the composition of digestive enzymes and ph conditions (Mulligan et al., submitted), which might result in differences in susceptibility to *Bt* toxins. Therefore, we investigated potential effects of *Bt* maize pollen expressing Cry1Ab or Cry3Bb1 on adult *C. carnea*. We selected a Cry3Bb1-expressing variety (MON 88017) and a Cry1Ab-expressing variety (Event Bt176) to ensure that adult lacewings were exposed to considerable amounts of Cry toxin throughout the experiments. A second bioassay was conducted with purified Cry1Ab and Cry3Bb1, where exposure was increased compared to the *Bt* protein concentration in pollen to ensure worst-case exposure conditions. This assay ensures a certain safety factor to reduce the likelihood that effects are missed in the laboratory but are present in the field. Furthermore, it allows general conclusions about the susceptibility to the *Bt* proteins independent from the maize variety.

## Results

### *Bt* maize pollen bioassay

#### Effects on life-table parameters

**Figure 1:**
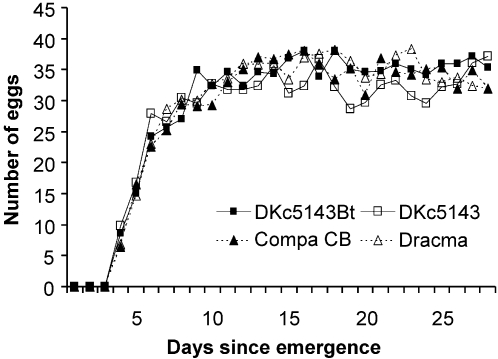
Mean daily fecundity of *Chrysoperla carnea* fed pollen from either of two *Bt* maize varieties or their respective non-transformed near isolines. Insects were provided 1 M sucrose solution together with pollen from: (a) DKc5143Bt expressing Cry3Bb1 (MON 88017), (b) the corresponding control DKc5143, (c) Compa CB expressing Cry1Ab (Event Bt176), and (d) the corresponding control Dracma. N = 30–34.

Mortality of the maize pollen-fed *C. carnea* in the course of the 28 days of the experiment remained very low (≤3%) (Table 1). All insects died within 7 days when provided with water only and were excluded from further analysis. The pre-oviposition period did not differ between the *Bt* and non-*Bt* pollen treatments (Mann-Whitney U-test; DKc5143Bt *vs*. DKc5143: U = 491, P = 0.377; Compa CB *vs*. Dracma: U = 422, P = 0.530) (Table 1). Daily fecundity was not significantly affected by *Bt*-transformation (RM-ANOVA; DKc5143Bt *vs*. DKc5143: F _1,65_ = 0.82, P = 0.367; Compa CB *vs*. Dracma: F _1,58_ = 0.10, P = 0.752) (Fig. 1). Student's t-tests revealed no statistical differences between *Bt* and non-*Bt* pollen treatments for total fecundity, fertility and dry weight of female and male lacewings at the end of the feeding experiment (P>0.3) (Table 1). None of the comparisons made between pollen from the two control varieties (DKc5143 *vs*. Dracma) revealed any significant difference (P>0.2) except that in the case of fertility the Student's t-test bordered significance (t = 1.98, P = 0.052). One to five pairs per treatment were discarded from statistical analyses since they laid no or only infertile eggs and were thus regarded as unmated.

**Table 1:** Impact of pollen consumption from Cry1Ab- or Cry3Bb1-expressing *Bt* maize or the corresponding non-transformed varieties on life-table parameters of adult *Chrysoperla carnea* and results from retrospective power analyses.

Pollen source	Number of pairs^a^	Survival (%)^b^	Pre-oviposition period (days±SE)^c^	Total fecundity (±SE)^d^	Egg hatching rate (%±SE)^d^	Adult dry weight	
						Female (mg±SE)^d^	Male (mg±SE)^d^
(a) DKc5143Bt (Cry3Bb1)	34	97.1	3.88±0.16	779.6±32.60	87.4±0.74	9.17±0.26	4.42±0.12
(b) DKc5143	33	97.0	4.03±0.15	735.1±36.58	86.8±1.08	9.25±0.27	4.51±0.17
(c) Compa CB (Cry1Ab)	31	100.0	3.71±0.14	779.3±28.68	82.5±1.80	9.30±0.25	4.64±0.16
(d) Dracma	30	98.3	3.83±0.15	769.6±46.52	82.5±1.79	9.33±0.26	4.55±0.18
Detectable difference (%)							
(a) *vs.* (b)		15.5	15.1	19.9	4.8	8.5	15.5
(c) *vs.* (d)		16.3	15.4	24.2	7.4	11.6	14.1

Pairs of *C. carnea* were fed 1 M sucrose solution together with pollen from one of four maize varieties: (a) DKc5143Bt (MON 88017), (b) the corresponding non-transformed control DKc5143, (c) Compa CB (Event Bt176), and (d) the corresponding non-transformed control Dracma. The experiment was terminated after 28 days. No statistical differences (P<0.05) were detected for (a) *vs.* (b), (c) *vs.* (d) and (b) *vs.* (d) for any of the parameters assessed.

Retrospective power analyses were conducted to calculate the detectable difference as percentage difference of detectable treatment means (a, c) relative to control means (b, d) (α = 0.05, power of 80%).

a Experiment started with n = 35 per treatment. Pairs producing no or only infertile eggs removed from analyses; ^b^Survival not analysed since mortality remained below 3% in all treatments. Retrospective power analyses based on log-rank test; ^c^Mann-Whitney U-test; ^d^Student's t-test.

Retrospective power analyses revealed that the detectable difference relative to the control means for the life-table parameters that were assessed varied strongly (Table 1). The lowest values obtained were for egg hatching rate, the highest for total fecundity. Values for the pre-oviposition period, survival, and weights of the females and males were in between.

#### Stability of *Bt* toxin in maize pollen

Results from enzyme-linked immunosorbent assays (ELISA) showed that the original concentrations of Cry3Bb1 and Cry1Ab proteins were 15.0±1.0 and 10.3±0.4 µg/g dry weight (mean±SE) in sub-samples of DKc5143Bt and Compa CB pollen, respectively, before the start of the feeding experiment. After 28 days storage at −20°C, *Bt* toxin contents did not decrease (Student's t-test; t = 0.97, df = 4, *P* = 0.386 for Cry3Bb1; t = 0.34, df = 4, P = 0.751 for Cry1Ab). After 3 days feeding exposure in the climate chamber, *Bt* toxin concentrations of pollen were not significantly different from fresh pollen (t = −0.75, df = 4, P = 0.495 for Cry3Bb1; t = 1.41, df = 4, P = 0.231 for Cry1Ab). No Cry toxin was detected in samples of non-transgenic maize pollen that were tested in parallel.

#### *Bt* toxin concentration in adult *C. carnea*

At the end of the experiment, the concentrations of Cry3Bb1 in female lacewings ranged from 0.05 to 2.35 with a mean of 1.38 µg/g dry weight in the Dkc5143Bt pollen treatment. In males, measured *Bt* toxin values were under the limit of quantification (0.022 µg/g dry weight) for 4 individuals and just above for 1 individual (0.027 ug/g). For females fed with Compa CB pollen, the concentrations of Cry1Ab were between 0.63 and 1.28 with a mean of 0.99 µg/g dry weight, and between 0.01 and 0.08 with a mean of 0.04 µg/g dry weight in males. No Cry toxin was detected in adults provided with non-transgenic maize pollen.

### Purified Cry toxin bioassay

#### Effects on life-table parameters

**Figure 2:**
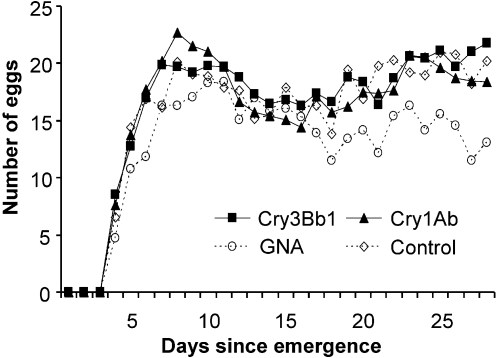
Mean daily fecundity of *Chrysoperla carnea* fed artificial diet containing insecticidal proteins. Per g dry weight, 150 µg Cry3Bb1, 120 µg Cry1Ab or 9 mg GNA (positive control) were incorporated. Pure diet served as a negative control. N = 33–35.

Adult survival was not affected by any of the insecticidal proteins tested (X^2^-test, P>0.1) compared to the control (Table 2). All adults provided with water only died within 7 days and were excluded from further analysis. Pair wise comparisons of the pre-oviposition period showed that the treatment containing either Cry3Bb1 or Cry1Ab did not significantly differ from the control at the adjusted α = 0.017 (Mann-Whitney U-test; Cry3Bb1: U = 437, P = 0.085; Cry1Ab: U = 448, P = 0.111) (Table 2). Adults fed GNA had a significantly longer pre-oviposition period than the adults fed control diet (U = 252, P<0.0001) (Table 2). Daily fecundity in the GNA treatment was significantly lower than in the control treatment (RM-ANOVA; F_1,67_ = 7.03, P<0.01) (Fig. 2). In contrast, no differences occurred when the Cry3Bb1 or Cry1Ab treatment was compared with the control treatment (P>0.1) (Fig. 2). Similar results were found for the total number of eggs laid per female (Dunnett test; Cry3Bb1: P = 0.893; Cry1Ab: P = 1.000; GNA: P = 0.039) (Table 2). None of the insecticidal proteins had an effect on fertility when compared with the control treatment (Dunnett test; Cry3Bb1: P = 0.998; Cry1Ab: P = 0.118; GNA: P = 0.992). At the end of the experiment, dry weights of Cry3Bb1- or Cry1Ab-fed females were similar when compared with those fed pure artificial diet (Dunnett test; Cry3Bb1: P = 0.870; Cry1Ab: P = 0.999). In contrast, in the GNA treatment, the dry weight of females was significantly lower than in the control treatment (P<0.0001). Similar results were found for males (Cry3Bb1: P = 0.992; Cry1Ab: P = 0.288; GNA: P = 0.003) (Table 2). One to three pairs per treatment were discarded from statistical analyses since they laid no or only infertile eggs and were thus regarded as unmated.

**Table 2:** Impact of feeding purified Cry3Bb1, Cry1Ab and GNA on life-table parameters of adult *Chrysoperla carnea*.

Treatment	Number of pairs^a^	Survival (%)^b^	Pre-oviposition period (days±SE)^c^	Toal fecundity (±SE)^d^	Eggs hatching rate (%±SE)^d^	Adult dry weight	
						Female (mg±SE)^d^	Male (mg±SE)^d^
Control	35	94.29	3.69±0.21	411.6±26.48	79.2±2.48	7.69±0.19	4.12±0.20
Cry3Bb1	33	93.75	3.82±0.13	431.5±25.61	80.4±1.56	7.98±0.66	4.07±0.28
Cry1Ab	33	90.91	3.88±0.14	415.8±26.22	83.9±2.26	7.67±0.21	3.83±0.13
GNA	34	89.71	4.62±0.20**	326.2±19.59*	80.5±1.94	6.42±0.30**	3.50±0.09**

Pairs of *C. carnea* were fed an artificial diet containing 150 µg Cry3Bb1, 120 µg Cry1Ab or 9 mg GNA (positive control) per g dry weight of artificial diet. Pure diet served as a negative control. The experiment lasted for 28 days.

Statistical comparisons were made for each of the insecticidal proteins with the control. Asterisks denote significant differences: ^*^ *P*<0.05, ^**^ *P*<0.01; ^a^Experiment started with n = 36 per treatment. Pairs producing no or only infertile eggs removed from analyses; ^b^Chi-square test; ^c^Mann-Whitney U-test; ^d^Dunnett test.

#### Stability of *Bt* toxin in artificial diet

The extraction efficiency of ELISA tests for Cry1Ab in the artificial diet was approximately 46%, with a measured mean (±SE) Cry1Ab concentration of 55.3±2.2 µg/g dry weight of artificial diet. After the 3-day feeding period, the concentration significantly decreased by 14% to 47.3±1.6 µg dry weight of artificial diet (Student's t-test; t = 2.90, df = 8, *P* = 0.020). For Cry3Bb1, 94% of the calculated concentration was detected, with a measured mean (±SE) concentration of 140.5±2.9 µg/g dry weight. After 3 days, a significant decrease of 13% to 112.8±3.3 was observed (t = 4.03, df = 8, *P* = 0.004). Apparently, Cry3Bb1 could be extracted more efficiently from the artificial diet than Cry1Ab, possibly because of different interactions and binding with components of the diet.

#### *Bt* toxin concentration in adult *C. carnea*

All adults tested positive for Cry1Ab or Cry3Bb1 toxin in the *Bt* treatments and no toxin was detected in adults fed pure diet. For insects fed with Cry1Ab-containing diet, the concentrations of Cry1Ab ranged from 2.12 to 4.45 with a mean of 3.05 µg/g dry weight in females, and from 0.25 to 1.77 with a mean of 1.23 µg/g dry weight in males. In the Cry3Bb1 treatment, the concentrations ranged from 0.71 to 19.9 with a mean of 15.2 µg/g dry weight in females and from 4.03 to 8.46 with a mean of 6.04 µg/g dry weight in males.

## Discussion

The pollen feeding bioassay did not show adverse effects on the fitness of adult *C. carnea* after ingestion of Cry3Bb1- or Cry1Ab-containing transgenic maize pollen when compared with pollen from the corresponding non-transformed maize varieties. Similarly, no difference between the two non-transformed maize varieties was detected for the assessed *C. carnea* life-table parameters. One exception to this was the small (ca. 5%) but almost significant difference (P = 0.052) in fertility, i.e. more eggs hatched in the DKc5143 pollen treatment compared to the Dracma treatment. This difference could be attributed to varying nutrient composition in the pollen of the different maize varieties. For example, Lundgren and Wiedenmann [13] reported that differences in the nutritional profile of pollen from different maize varieties were correlated to the mortality of larvae and adults of the pollen feeding ladybird beetle *Coleomegilla maculata* DeGeer (Coleoptera: Coccinellidae).

Our ELISA measurements have shown that *C. carnea* females ingested considerable Cry3Bb1 and Cry1Ab protein concentrations during the 28 days of *Bt*-pollen-feeding, while males contained at least 25 times lower amounts of toxin. In contrast to females, which need carbohydrates as energy source and pollen as protein source for reproduction, males are likely to survive well on carbohydrates alone. Thus the males most likely consumed mainly sugar solution and only small amounts of maize pollen, which explains the low *Bt* toxin concentrations measured.

The toxin concentrations measured in fresh pollen from Compa CB (Event Bt176) in our study were higher than previously reported from field samples. Since *Bt* maize based on this event is not grown anymore, we need to compare the Cry1Ab concentrations with the currently grown *Bt* maize varieties, that contain the events Bt11 and MON810. Field collected pollen from these *Bt* varieties contains Cry1Ab concentrations less than 90 ng/g dry weight pollen [14], which is by 2 orders of magnitude lower that the pollen used in our study. The concentration of Cry3Bb1 in pollen of DKc5143Bt (MON 88017) was slightly below field expression levels ranging from 17 to 32 µg/g dry weight [28]. Different expression levels may be due to the growing conditions of our maize plants or the use of different varieties. In addition, ELISA results commonly vary between studies conducted with different kits, extraction methods and purified standard proteins used. There was no apparent degradation of the Cry toxins in maize pollen during the 3-days exposure to the lacewings. The biological activity of Cry3Bb1 and Cry1Ab in the pollen samples used in the feeding bioassays was confirmed by sensitive insect bioassays using larvae of *Leptinotarsa decemlineata* (Say) (Coleoptera: Chrysomelidae) and *O. nubilalis*, respectively, in our laboratory (Li et al. unpublished data). For Event Bt176 this is consistent with previous studies [33], [34]. Consequently, *C. carnea* adults were exposed to a constant level of active Cry toxins when feeding on *Bt* maize pollen in our experiment. Despite the fact that our feeding experiment lasted more than twice as long as the pollen shedding period in maize fields that usually spans 5–8 days with a maximum of 14 days [7], [35], no adverse effects were observed. This indicates that adult *C. carnea* are not detrimentally affected when consuming pollen from the two tested *Bt* maize varieties.

To draw a general conclusion about the sensitivity of adult *C. carnea* to Cry1Ab or Cry3Bb1 independent from the plant background, and to address the fact that our ELISA measurements of the pollen revealed a lower Cry3Bb1 concentration than reported from field collected pollen, a bioassay using activated and purified toxins, incorporated into artificial diet, was carried out. A concentration about 10 times higher than that measured in maize pollen was used to achieve a worst-case exposure scenario and to add a safety margin and thus increase the certainty of the hazard assessment [4], [5]. No adverse impact of the two Cry toxins was found on any of the tested *C. carnea* life-table parameters. ELISA measurements confirmed that adult lacewings contained higher concentrations of Cry toxins when fed toxin-containing diet compared to *Bt* maize pollen due to the higher concentrations provided in the artificial diets. Similar to the *Bt* pollen bioassay, males contained lower amounts of *Bt* toxin than females. The difference, however, was only 2.5 times. In contrast to the pollen bioassay, no additional carbohydrate source was present and the males consumed the same diet as the females. Even though ELISA tests revealed a significant degradation of the Cry toxins in the artificial diet the concentration measured after the 3 day feeding exposure was still more than 8 fold higher than in pollen. Bioactivity of both purified toxins was confirmed by sensitive insect bioassays with the same batch of Cry3Bb1 and Cry1Ab. Therefore, we can conclude that adult *C. carnea* are not sensitive to Cry1Ab or Cry3Bb1 at concentrations that are about 8–10 fold higher compared to the concentrations expressed in the pollen of the two tested *Bt* varieties.

In the current study, the mean daily fecundity per female reached over 30 eggs one week after emergence in any treatment of the pollen feeding experiment. A lower oviposition level of about 15–23 eggs per day was reached in the artificial diet experiment for females fed control diet or diet containing Cry toxin. As expected, females in the positive control treatment, i.e. fed with GNA containing diet, reached an oviposition rate of less than 15 eggs per day. Thus, in both experiments the average daily fecundity in the control treatments was above 15, one validation criteria of the standardized protocol to evaluate effects of pesticides on *C. carnea* [36]. In addition, the fertility in any treatment of the two experiments was also higher than the threshold value of 70% defined by Vogt et al. [36]. Thus, the experimental procedures used in our study appeared to be appropriate and valid for assessing the hazard of orally active insecticidal proteins on adult *C. carnea*.

When performing statistical tests, one faces the risk of committing type II errors, i.e. failing to reject a false null hypothesis. In other words, an existing difference might not be found statistically because the power of the test was too low to detect it. However, our purified toxin bioassay was able to detect effects caused by the positive control (GNA). This means that our experimental system was sensitive enough to show a 25% lower pre-oviposition period, a 21% lower total fecundity and a 15% lower dry weight of adult lacewings in the GNA treatment compared to the control diet. Retrospective power analyses were thus not conducted for this bioassay. Due to the absence of statistical differences for any of the *C. carnea* life-table parameters assessed in the maize pollen bioassay, retrospective power analyses were conducted to calculate the difference between control and treatment mean in our bioassay set-up separately for the two *Bt* maize and non-transformed maize plant pairs that could have been detected with a power of 80% at a 5% type I error rate. These analyses revealed that the detectable differences were (with one exception) lower than the 20% effect size that is usually set for regulatory risk assessment studies [37]. Consequently, effects above this threshold should have been detected, if present.

Despite the fact that *Bt* maize pollen has raised much attention as an exposure route of Cry toxins to non-target arthropods, especially in regard to potential effects on non-target butterflies [34], [35], [38], only few studies addressed potential impacts of *Bt* maize pollen on natural enemies. *C. maculata* larvae and adults were fed with Cry3Bb1 containing *Bt* maize pollen, but no effects on any life-table parameter were observed [26], [39], [40]. Ahmad et al. [40] also reported no effects of Cry3Bb1 protein expressed in *Bt* maize pollen on two carabid species, *Harpalus caliginosus* F. and *Harpalus pensylvanicus* DeGeer. Similarly, Cry1Ab-expressing *Bt* maize pollen revealed no detrimental impact on *C. maculata* larvae [10], [13]. Pilcher et al. [10] also fed larvae of *Orius insidiosus* Say (Heteroptera: Anthocoridae) and *C. carnea* with pollen from Cry1Ab expressing *Bt* maize (Event Bt176) and did not find effects on several life-table parameters. Zemek & Vavrova [41] reported no effects of Cry1Ab expressing maize (MON 810) pollen on the predatory mite *Typhlodromus pyri* Scheuten (Acari: Phytoseiidae). In contrast to the above studies, feeding on pollen from *Bt* maize (Bt 11) did cause subtle effects on the predatory mite *Neoseiulus cucumeris* (Oudemans) (Acari: Phytoseiidae) when compared to non-*Bt* maize pollen [42]. These effects, however, were most probably not caused by the Cry toxin but by unidentified differences in pollen nutrition since the predatory mites remained unaffected when feeding on *Bt* maize-fed spider mites which contained many-fold higher concentrations of the toxin compared to pollen [42].

Overall, our results confirm the earlier reported specificity of the two Cry proteins and the lack of direct detrimental non-target effects under laboratory and field conditions [27], [28], [43]–[45]. In particular, a recent meta-analysis on field studies consistently failed to detect population changes of *Chrysoperla* spp. in *Bt* crops [45].

The present laboratory studies show that adult *C. carnea* appear to be non-sensitive to Cry1Ab and Cry3Bb1 and that feeding on *Bt* maize pollen containing either of the two proteins poses a negligible risk to this beneficial species. This includes the currently grown Coleoptera-resistant *Bt* maize event MON 863 and Lepidoptera-resistant varieties that are based on the events MON 810 and Bt11 which contain substantially less Cry1Ab toxin in the pollen when compared to the Event Bt176-based variety used in this study. Furthermore, the information derived from our study will be applicable to the risk assessment of other crops and maize varieties expressing the same Cry proteins.

## Materials and Methods

### Insects

*C. carnea* were collected in Bolligen near Berne (Switzerland) in 1993 and since then maintained in the laboratory without introductions of field-collected insects [29]. Eggs collected from the colony were kept in a climatic chamber at 22±1°C, 75±5% RH and 16∶8 h L∶D. After hatching, the larvae were fed with eggs of *Ephestia kuehniella* Zeller (Lepidoptera: Pyralidae) provided by Biotop (Valbonne, France). When the larvae had reached the second instar, they were kept individually in plastic tubes (5.5 cm high, 1.5 cm diameter) containing *ad libitum E. kuehniella* eggs until pupation. Newly emerged (<24 h after emergence) adults were used for all experiments.

### Plants

Two transgenic maize varieties, Compa CB (Event Bt176, Syngenta, Stein am Rhein, Switzerland) and Dkc5143Bt (Event MON 88017, Monsanto Company, St. Louis, USA) and their corresponding non-transformed near isolines Dracma and Dkc5143, respectively, were used for the experiments. Compa CB plants express a synthetically modified Cry1Ab gene from *B. thuringiensis* ssp. *kurstaki* HD-1 targeting Lepidoptera. Cry1Ab expression is driven by the constitutive PEPC promoter as well as a pollen-specific promoter [46]. DKc5146Bt plants express a synthetically modified gene from wild-type *B. thuringiensis* ssp. *kumamotoensis* EG4691 selectively targeting Coleoptera such as corn root worms, *Diabrotica* spp. [47]. Expression of the *cry3Bb1* gene is driven by the constitutive e35s cauliflower mosaic virus promoter [28].

Maize plants were grown individually in plastic pots (12 l) in a glasshouse. Before sowing, 40 g slow release fertilizer (Osmocote Exact, 16% N: 11% P_2_O_5_: 11% K_2_O, Scotts UK Professional, Bramford, UK) was mixed into the soil in each pot. Plants were fertilized weekly with 400–800 ml of a 0.2% aqueous solution of Vegesan standard (80 g N, 70 g P_2_O_5_ and 80 g K_2_O per liter, Hauert HBG Dünger AG, Grossaffoltern, Switzerland) and watered as required. To ensure comparable conditions, plants from all four maize varieties were grown together in the same glasshouse from July to October 2005.

### Pollen collection

When plants reached anthesis, each tassel was confined in an air-permeable cellophane bag (19.5×37.5 cm, Celloclair AG, Liestal, Switzerland). A small hole was cut in the bottom of the bag to collect pollen every day. Collected maize pollen was air dried at room temperature for 24 h and subsequently sifted using a mesh size of 0.2 mm to remove anthers and contaminants [34]. To avoid cross-contamination, different sieves were used for pollen from the different maize varieties. Pollen collected from 50 plants of each variety was pooled and stored at −80°C for about 8 months until use.

### Purified toxins

Lyophilised Cry1Ab was purchased from M. Carey (Dept. Biochemistry, Case Western Reserve University, Cleveland, OH, USA). The Cry1Ab protoxin from *B. thuringiensis* subsp. *kurtsaki* HD-1 was expressed as a single gene product in *Escherichia coli*. Inclusion bodies containing Cry1Ab protoxin were dissolved and trypsinised and the Cry1Ab toxin was isolated using high-performance liquid chromatography. The bioactivity of Cry1Ab was confirmed in a sensitive insect bioassay using neonate *O. nubilalis* larvae. Toxin solutions were mixed into artificial diet and fed to neonate larvae for 7 days. The EC_50_ (toxin concentration resulting in 50% weight reduction) was estimated to be 5 ng/ml diet (data not shown). Cry3Bb1 toxin (provided by Monsanto Company) was produced by fermentation of *E. coli* containing the Pmon72735 expression plasmid and purified using SDS-PAGE/Densitometry. Insect bioassays using Colorado potato beetles *L. decemlineata* conducted at Monsanto showed that the LC_50_ (concentration resulting in 50% mortality) of our batch was 0.4 µg/ml diet when neonate larvae were fed for 1 week with toxin containing artificial diet. Snowdrop lectin (*Galanthus nivalis* agglutinin, GNA) was obtained from E. van Damme (Ghent University, Belgium). A detailed description of the isolation from snowdrop bulbs is given by Van Damme et al. [48].

### Bioassay conditions

All experiments were conducted in a climatic chamber at 22±1°C, 75±5% RH and 16∶8 h L∶D. Single pairs of *C. carnea* adults were confined in transparent plastic cylinders (6.0 cm diameter, 8.5 cm high). Each plastic cylinder was covered with a lid which contained a 4 cm opening to allow ventilation. Between cylinder and lid, a layer of cotton gauze prevented escapes and served as oviposition substrate. Water was provided by a cotton dental wick, which was positioned through a 1 cm hole at the bottom of each container. The cylinders were placed closely over a water reservoir so that the wicks were submerged and a continuous water supply was ensured. Water in the reservoir was replaced once a week. Since mating of adult *C. carnea* generally occurs during the first few days and remating is not required to ensure egglaying throughout the 28-day test period [49], males that died during the experiments were not replaced.

### *Bt* maize pollen bioassay

Preliminary experiments with adult *C. carnea* showed that maize pollen (Dracma) provided together with 1 M sucrose solution resulted in similar reproduction and survival as an artificial diet consisting of sucrose, brewer's yeast and water (in proportions 7∶4∶4), which is considered as highly nutritious for adult *C. carnea* [49], [50].

The sex of freshly emerged adults of *C. carnea* was determined and randomly selected pairs were kept in the plastic cylinders described above. *Ad libitum* maize pollen and 1 M sucrose solution were offered separately in small plastic dishes (2.5 cm diameter, 0.7 cm high), which were placed on the bottom of the test containers and replaced every 3 days. In addition, a water only treatment served as non-food control.

Thirty-five pairs of adult *C. carnea* were tested for each of the 4 pollen treatments and 20 pairs for the water only treatment. The pre-oviposition period (mean number of days from emergence to the first oviposition), survival, daily and total fecundity (number of eggs laid) and fertility (egg hatching rate) were recorded and/or calculated based on daily observations. After the daily counting, eggs were removed from the test-containers. Fertility of the eggs was assessed for each female at the beginning of oviposition, as well as in the middle of the experiment and towards the end. Collected eggs were placed in a separate container together with their oviposition substrate (gauze) and *ad libitum E. kuehniella* eggs to minimize cannibalism of hatching larvae. Seven days later, hatched larvae were counted. For each female, at least 40 eggs were checked. After 28 days (ca. twice as long as fresh maize pollen is usually present in field [35]), the feeding-experiment was stopped. Five males and females from the *Bt* treatments were randomly selected to confirm the presence of Cry toxins in the insect bodies using ELISA as described below. The remaining adults were dried at 50°C for 4 days and the dry weight was determined.

During the experiment, all pollen was stored in a −20°C freezer. In order to determine the stability of the Cry3Bb1 and Cry1Ab proteins under those storage conditions, 5 pollen sub-samples were taken from both *Bt* varieties before the experiment and after 28 days in the freezer. In addition, toxin degradation between the feeding occasions was measured by taking 5 sub-samples of maize pollen before and after 3 days feeding-exposure. All pollen sub-samples were stored at −80°C until ELISA analysis.

### Purified Cry toxin bioassay

The second bioassay using purified Cry toxins was conducted in a similar way. Pairs of *C. carnea* were kept individually and assigned to 1 of 4 dietary treatments: (i) artificial diet (described above) with purified Cry3Bb1 protein; (ii) artificial diet with purified Cry1Ab protein; (iii) artificial diet with purified snowdrop lectin (GNA; positive control); (iv) pure artificial diet (negative control). The concentrations of Cry3Bb1 and Cry1Ab protein in diets were calculated to be 150 and 120 µg/g dry weight, respectively, which was about 10 times higher than the *Bt* concentration detected in maize pollen from DKc5143Bt and Compa CB based on previous ELISA analysis. The concentration of GNA in the diet was 1% (weight/volume), which was equal to 9 mg/g dry weight. GNA was used as a positive control since a previous study has revealed that this compound affects several life-table parameters of adult *C. carnea* after ingestion at this particular concentration (Li & Romeis, submitted). All the diets were prepared 3 days before initiation of the study and were stored at −80°C until use.

For each dietary treatment, 36 pairs of newly hatched adult *C. carnea* were tested. The diets were smeared on one end of a green plastic label (9 cm×1.6 cm) which was placed on the bottom of the test container. All diets were provided *ad libitum* and replaced 3 three days. Water was provided as described above. Similar to the maize pollen bioassay, the pre-oviposition period, survival, daily and total fecundity and fertility were recorded. After 28 days, all surviving adults were lyophilized and weighed (dry weight). Ten females and males of each *Bt* treatment were randomly selected for measuring Cry toxin using ELISA.

The concentrations of Cry3Bb1 and Cry1Ab in the artificial diets were measured before and after the 3 days of feeding exposure to check potential degradation of the *Bt* toxins. All samples were stored at −80°C until ELISA analysis.

### ELISA measurements

The concentrations of Cry3Bb1 and Cry1Ab in pollen, artificial diet and lacewings were measured using double-antibody sandwich enzyme-linked immunosorbent assays (DAS-ELISA) from Agdia (Elkhard Indiana, USA). Prior to analysis, lacewings were washed in phosphate buffered saline Tween (PBST) buffer (provided in the kit) to remove any *Bt* toxin or *Bt* maize pollen from their outer surface.

After adding PBST to the samples at a ratio of at least 1∶10 (mg sample∶µl buffer) in 2 ml microreaction tubes, a 5 mm tungsten carbide bead was added and the samples were macerated for 3 min at 30 Hz in a mixer mill MM300 (Retsch, Haan, Germany) fitted with 24 tube-adapters (Quiagen, Hombrechtikon, Switzerland). After centrifugation and appropriate dilution of the supernatants, the ELISA was performed according to the manufacturer's instructions. The measured OD values were calibrated using a range of Cry1Ab and Cry3Bb1 standards made from purified toxin solutions.

### Data analyses

In the maize pollen experiment, statistical comparisons were made between pollen from *Bt* maize and that from the corresponding non-transformed control variety. In addition, means of the two non-transformed varieties were compared to test for possible variety effects. Thus 3 pair wise comparisons were made. For the purified toxin bioassay, comparisons were conducted for each protein with the control using Dunnett test wherever possible, i.e. when the assumptions of parametric test (normal distribution of residues and homogeneity of error variances) were met (total fecundity, fertility and adult dry weight). For the other parameters (percentage survival, pre-oviposition period), each treatment was compared separately with the control. Bonferroni correction was applied to correct for 3 pair wise comparisons leading to an adjusted α = 0.017.

Mortality of the maize pollen-fed *C. carnea* remained very low during the experiment and was thus not analysed. In the purified toxin bioassay, means of the toxin treatments were compared to the control using Chi-square tests. For the pre-oviposition period, means were compared using Mann-Whitney U-test in both experiments since the assumptions for parametric analyses were not fulfilled. In the maize pollen bioassay, data on total fecundity, fertility (percentage of hatching eggs) and adult dry weight were analyzed using Student's t-test. Fertility data were transformed by arcsine√(x) and dry weights of males by log(x) before analysis. Daily fecundity was analyzed using repeated-measures (RM) ANOVA.

ELISA measurements of fresh and old *Bt* maize pollen were compared using Student's t-test.

Statistical analyses were conducted using the software package STATISTICA (version 7, StatSoft, Inc., Tulsa, USA). To avoid committing type II errors, i.e. failing to reject a false null hypothesis, retrospective power analyses were conducted on non-significant results (*P*>0.05) in the maize pollen bioassay using PASS (Version 2005, NCCS, Kaysville, Utah, USA). Using the observed control means and standard deviations and the true sample sizes, the detectable differences (percentage difference of detectable treatment means relative to control means) were calculated for α = 0.05 and a power of 80% [51]. Power analyses were not conducted for the purified toxin bioassay since it included the insecticidal protein GNA as a positive control. Effects observed for GNA ensured that the bioassay was sensitive enough to detect significant effects of the tested Cry proteins, if present (Li & Romeis, submitted).
